# The Whale Optimization Algorithm Approach for Deep Neural Networks

**DOI:** 10.3390/s21238003

**Published:** 2021-11-30

**Authors:** Andrzej Brodzicki, Michał Piekarski, Joanna Jaworek-Korjakowska

**Affiliations:** Department of Automatic Control and Robotics, AGH University of Science and Technology, 30-059 Cracow, Poland; brodzicki@agh.edu.pl (A.B.); jaworek@agh.edu.pl (J.J.-K.)

**Keywords:** whale optimization algorithm, optimization, deep learning, neural networks, hyperparameters

## Abstract

One of the biggest challenge in the field of deep learning is the parameter selection and optimization process. In recent years different algorithms have been proposed including bio-inspired solutions to solve this problem, however, there are many challenges including local minima, saddle points, and vanishing gradients. In this paper, we introduce the Whale Optimisation Algorithm (WOA) based on the swarm foraging behavior of humpback whales to optimise neural network hyperparameters. We wish to stress that to the best of our knowledge this is the first attempt that uses Whale Optimisation Algorithm for the optimisation task of hyperparameters. After a detailed description of the WOA algorithm we formulate and explain the application in deep learning, present the implementation, and compare the proposed algorithm with other well-known algorithms including widely used Grid and Random Search methods. Additionally, we have implemented a third dimension feature analysis to the original WOA algorithm to utilize 3D search space (3D-WOA). Simulations show that the proposed algorithm can be successfully used for hyperparameters optimization, achieving accuracy of 89.85% and 80.60% for Fashion MNIST and Reuters datasets, respectively.

## 1. Introduction

Deep learning is currently one of the most popular and rapidly developing section of artificial intelligence and is mostly based on advanced and sophisticated neural network architectures which are widely used for tasks including image segmentation, classification, signal analysis, data investigation and modelling [1,2]. One of the most challenging parts while deploying deep neural network architecture is the training process which is responsible for achieving the highest score while there is a certain inefficiency due to very long training time required. Obtaining the most accurate deep neural network (DNN) within a reasonable run-time is still a huge challenge. Furthermore, training the network requires setting a few hyperparameters such as number of epochs, batch size, learning rate or optimizer which generates another non-trivial optimisation problem, as it is basically an optimisation of an optimisation.

Meta-heuristic algorithms such as artificial bee colony, particle swarm optimization, genetic algorithm and differential evolution have a great potential for optimising both network architectures and training parameters [3]. They have already been applied in many fields where finding optimal solution was beneficial, like power systems, applied mathematics, IoT, cryptography, cloud computing as well as automatics (e.g., tuning controllers) [4]. Therefore, we decided to utilise this approach in deep learning by optimising neural network hyperparameters. In particular, the use of artificial intelligence (deep neural networks in the first place) and thus optimization methods takes place in many sensor fusion algorithms for object detection and classification in fields like Autonomous Vehicles (AV) or Unmanned Aerial Vehicles (UAVs) [5,6,7]. The key task in such systems is to train the deep architecture in such a way that from the data from many sensors (such as cameras, radars or lidars) it is possible to create a model of the environment with the assumed high accuracy. Moreover, in the great majority, embedded intelligence has to perform in a resource-constrained environment, such as low-power edge devices or smart sensors. Therefore it is vital to find the optimum set of hyperparameters for the models to work accurately with the minimum energy consumption on the target platforms.

One of the latest examples of meta-heuristic algorithms, that has not yet been widely explored, is the Whale Optimisation Algorithm (WOA), presented in 2016 by Seyedali Mirjalili and Andrew Lewis [8]. A systematic review of contemporary implementations can be found in [9].

The novelty of this work can be summarised as follows:

- To the best of our knowledge this is the first paper that uses Whale Optimisation Algorithm for deep neural networks training hyperparameters optimisation.
- We compare our solution with the widely used optimization algorithms including Grid and Random Search.
- We have tested this implementation on popular deep learning databases and task examples to assess and evaluate the model.
- We verified the possibility of using continuous Whale Optimisation Algorithm in a discrete choice problem by implementing a transfer function.
- We have adjusted the WOA algorithm and implemented a third dimension feature analysis to the original WOA algorithm to utilize 3D search space (3D-WOA).

This paper is organized in five sections as follows. Section 1 presents briefly meta-heuristic algorithms, importance of training hyperparameters optimisation and covers information about the WOA applications in the DNN field. Section 2 contains justification of the chosen methods and approach, describes inspirations of the WOA algorithm, its mathematical background, context of planned experiments as well as our 3D-WOA implementation. Section 3 shows in detail testing conditions, benchmark cases as well as conducted tests and its results. We also mention there software and hardware environment used is all experiments. Section 4 includes considerations on the results achieved compared to related works. Finally, Section 5 covers summary of research work and suggests possible further study directions.

### Related Works

Different algorithms have been proposed to optimize the architecture and parameters of deep neural networks [10]. The most widely used include: Grid Search as a basic method which performs an exhaustive search on the parameters set and Random Search as an improvement of the previous method by introducing randomized search over hyper-parameters from certain distributions over possible parameter values [10]. As the problem of optimizing neural networks has not been solved yet, various evolution-based approaches have been proposed. They can be divided into a few subcategories including the most popular based on swarm and genetic algorithms. Swarm Intelligence is a category of algorithms, that consists of many individual agents that independently explore the search space, but in the same time have the ability to communicate with one another, for example informing about the current best solution. The inspiration for those methods is often taken from nature—such as flocks of birds, ant colonies or in our case whales. The examples of the swarm-based algorithms used for deep learning parameter optimisation have been described in papers [11,12,13]. In [11] Authors have used the Particle Swarm Optimisation (PSO) algorithm to select architecture parameters for a convolutional neural network to classify CIFAR dataset. In paper [12], less popular algorithms like grasshopper and greywolf were used to adjust computation parameters of a network dedicated to assess a heating load (HL) of residential buildings. Finally, in [13] Authors have modified two meta-heuristic algorithms (tree growth and firefly algorithm) and tested their performance on tuning deep learning training hyperparameters for the classification task which has been tested on the widely used MNIST dataset.

On the other hand, evolutionary algorithms, or more specifically genetic algorithms, try to create the best solution for the task by implementing operations analogical to gene mutation, crossover and selection. They are creating the best candidate from the initial random population using methods drawn from evolution. The solutions based on genetic algorithms (GA) have been proposed in [14,15,16,17]. One of the first research that successfully utilises genetic algorithms for hyperparameters tuning was described in 2015 [14]. The authors implemented the solution that automatically optimises parameters of a convolutional neural network based architecture, independently for different tasks. In paper [15] authors state that genetic algorithms with enfixed-length chromosomes may fail in deep learning hyperparameters optimisation. Therefore they propose a variable length algorithm that achieves good results in automatic parameter tuning. Another interesting approach was presented in paper [16]. The authors tried to reduce the network training time by choosing the right hyperparameter set. Like in our research, they verified it on a very simple network, trained on MNIST dataset, typically used for this kind of benchmarks. Finally, in paper [17] authors performed an in-depth study of comparing different methods used for another popular dataset—CIFAR-10. They conclude their research by choosing a hybrid of genetic algorithms with local search method as the best solution (an approach similar to Whale Optimisation Algorithm which has both exploration and exploitation phase). Another popular algorithm, that draws some inspiration from both nature and genetics is a Cuckoo Search approach. Although mostly used in classical optimisation tasks, it finds use in deep learning as well. It has been used for selecting optimal features and classifiers in a simple classification task [18].

Among many applications already using WOA there are some that utilize it in other deep learning field. Dixit et al. proposed applying Whale Optimization Algorithm in convolutional neural network architecture in the texture classification task [19]. WOA has been employed for optimizing values of the filters in convolution layers as well as for weights and biases optimization in fully-connected ones. Presented model outperformed existing methods for the given datasets. Ibrahim Aljarah et al. proposed WOA-based trainer for multi-layer perceptron (MLP) network [20] and tested it on twenty different datasets, compared with backpropagation algorithm and six evolutionary methods. WOA was able to outperform other algorithms in terms of not only accuracy but also convergence. Similar hybrid WOA-MLP architecture has been proposed by Zakaria Alameer et al. [21], where it improved the performance in forecasting gold price fluctuations, compared to other algorithms. Therefore, we decided to focus solely on training hyperparameters, as this field still requires further research.

Whale Optimisation Algorithm, which we test in this research is a type of a swarm-based algorithm. To the best of our knowledge, this is the first attempt to use it for optimising typical deep neural networks training hyperparameters.

## 2. Materials and Methods

In this section, we describe the WOA algorithm presented in [8]. We explain its main ideas and mathematical background. Furthremore, we explain our own implementation of a 3-dimensional version of the WOA algorithm and present the method in context of deep neural network hyperparameters optimization problem. The experiments have been conducted on widely-used machine learning algorithms including Fashion MNIST and Reuters which are typically used to prove new concepts in machine learning an ahve been described in Section 3. We have also describing the improvements that were needed to adapt the basic WOA algorithm for the deep learning hyperparameter optimisation.

### 2.1. Whale Optimisation Algorithm

#### 2.1.1. Nature-Inspired Solution

A vast majority of meta-heuristic algorithms have been inspired by nature. Similarly, Whale Optimisation Algorithm has been implemented after a careful analysis of whales’ behavior, in particular the intelligent hunting behavior of humpback. Whales are extraordinary creatures not only because they are the biggest mammals in the world but also due to the fact that they are highly intelligent and have developed social behaviors.

**Figure 1:**
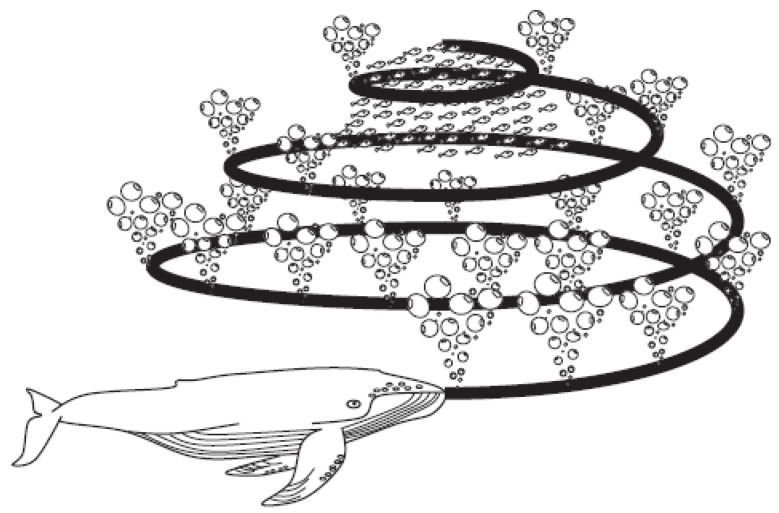
Visualization of bubble-net feeding hunting method. The humpback swims in a spiral below the prey, creating bubbles that trap them [8]. Reproduced with permission from Elsevier publisher, licence no. 5198660218147.

Direct inspiration for a Whale Optimisation Algorithm was special hunting method of humpback whales which is called bubble-net feeding method. It is a hunting method unique for this species [8], consisting in diving under a shoal of small fish or krill and then approaching the surface on a spiral trajectory around the shoal while creating and releasing air bubbles. The visualization of bubble-net feeding hunting method has been presented in Figure 1.

#### 2.1.2. Mathematical Model of Spiral Bubble-Net Feeding Behavior

The mathematical model is directly inspired by a humpback’s bubble-net feeding method and has been proposed in [8] by Seyedali Mirjalili and Andrew Lewis. The starting point of the mathematical model and the first element of the hunting method described in the previous section is finding the location of the prey by a herd. After choosing the best first search agent (after calculating the fitness of each search agents), others pursue his position by updating their positions according to the given steps:

(1) $$\vec{D}=\left|\vec{C}\cdot \vec{X^{*}}\left(t\right)-\vec{X}\left(t\right)\right|$$

(2) $$\vec{X}\left(t+1\right)=\vec{X^{*}}\left(t\right)-\vec{A}\cdot \vec{D}$$

(3) $$\vec{A}=2\vec{a}\cdot \vec{r}-\vec{a}$$

(4) $$\vec{C}=2\cdot \vec{r}$$

where *t* is the current iteration, $X^{*}$ is the position vector of the best solution, $\vec{X}$ is the position vector, $\vec{a}$ is linearly decreased vector over the iterations, $\vec{r}$ is a random vector in [0, 1].

Achieving different new positions (X,Y) in the neighbourhood of the best agent $\left(X^{*},Y^{*}\right)$ is possible by updating values of $\vec{A}$ and $\vec{C}$ vectors according to Equations (3) and (4). What is worth mentioning is that this model can be extended to the *n* dimensional search space which we benefit from in our research by extending the space to three-dimensional one.

**Figure 2:**
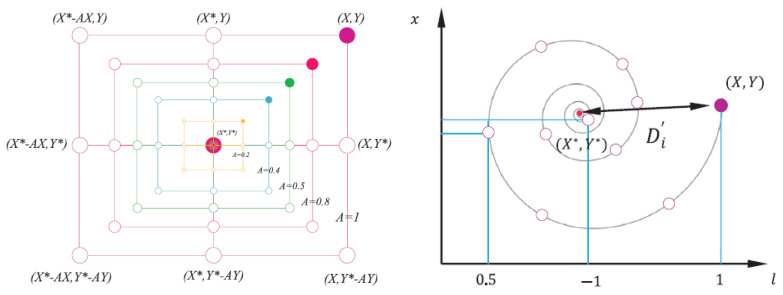
Bubble-net hunting attack model consists of two stages: shrinking encircling mechanism (**left**) and spiral updating position (**right**), chosen in semi-random order [8]. Reproduced with permission from Elsevier publisher, licence no. 5198660218147.

The main part of the bubble-net hunting method is the attack itself. In WOA it is called *exploitation phase* and is divided into two stages: *shrinking encircling mechanism* and *spiral updating position*. Ref. [8] First behaviour is mathematically represented by a vector $\vec{a}$ with decreasing value over the iterations and the second by introducing special equation to model spiral movement of the whales (Equation (5)). In fact, when hunting, both of these behaviors occur simultaneously, as the whales move towards the surface in a spiral trajectory while shrinking its diameter. Therefore to imitate this, Whale Optimisation Algorithm assumes 50% probability to update position by utilizing either the first or the second behaviour (Equation (7)).

(5) $$\vec{X}\left(t+1\right)=\vec{D^{\prime }}\cdot e^{bl}cos\left(2\pi l\right)+\vec{X^{*}}\left(t\right)$$

(6) $$\vec{D^{\prime }}=\left|\vec{X^{*}}\left(t\right)-\vec{X}\left(t\right)\right|$$

where *b* is a constant for defining the shape of the logarithmic spiral, *l* is a random number in [−1,1]. Figure 2 presents bubble-net hunting attack split into two separate movements in the mathematical model.

(7) $$\vec{X}\left(t+1\right)=\left\{\begin{array}{c} \vec{X^{*}}\left(t\right)-\vec{A}\cdot \vec{D}\;\mathrm{if}\;p<0.5\\ \vec{D^{\prime }}\cdot e^{bl}cos\left(2\pi l\right)+\vec{X^{*}}\left(t\right)\;\mathrm{if}\;p\geq 0.5 \end{array}\right.$$

Whale Optimisation Algorithm introduces also another mechanism called *exploration phase*. Its main role is to explore the search space away from the current best solution in order to potentially find a better one. Therefore, WOA has a global search ability. Mathematically, this is done by updating the position (X,Y) based on the random vector $\vec{A}$ (Equation (9)).

(8) $$\vec{D}=\left|\vec{C}\cdot \vec{X_{rand}}-\vec{X}\right|$$

(9) $$\vec{X}\left(t+1\right)=\vec{X_{rand}}-\vec{A}\cdot \vec{D}$$

where $\vec{X_{rand}}$ is randomly chosen position vector for a current iteration.

Whale Optimisation Algorithm steps can be summarized as:

- Start with a random initialization of the search agents and choose the best, first solution.
- In every step, update the position based on a different equation, depending on the value of *p* and $|\vec{A}|$
  – Utilize spiral movement by updating current position by Equation (5)
  – Introduce *exploration* or *exploitation* phase by updating current position by Equation (9) or Equation (2), respectively
- Check search space constraints and update best solution if there is a better one found
- Finally, algorithm loop is terminated by the maximum number of iterations (default criterion).

### 2.2. WOA Algorithm for Deep Neural Network Optimization

In our research we take advantage of Whale Optimization Algorithm for deep neural network architecture training and hyperparameters selection to find the best set of parameters. To specify our problem, we define three things—mainly the cost function, parameter search space and how we constrain it.

To test and confirm the WOA performance and effectiveness in optimizing the training parameters we run it on a few simple benchmark optimization problems and compared the performance with other conventional optimization algorithms such as random search and grid search. Data sets and labels were taken from the UCI machine learning repository. They included Fashion-MNIST images classification as well as Reuters text data classification. Fashion-MNIST is a replacement of a popular benchmark MNIST dataset. We decided to use Fashion-MNIST because, as its authors suggest, the original MNIST is too simple for modern machine learning tasks, and overused in too many research [22]. Fashion-MNIST dataset consist of 28 × 28 grayscale images of clothes, each belonging to one of the 10, equally numerous classes. There are 60,000 images in a train set and 10,000 in test. The second one—Reuters dataset is often used for verifying simple text interpretation. Its latest version, available in Keras, consists of 8982 train and 2246 test newspaper articles, divided into 46 classes based on the topic. In our experiments, we have retained the original splits into train and test subsets to allow an easy replication of our results. GridSearch and RandomSearch methods, as well as WOA-based approach were run on train set with a 3-fold random cross-validation in each of the test runs. Then, final results were evaluated on an unchanged, fixed test set, provided by the dataset authors.

To solve this basic tasks we used a simple deep learning architectures build from fully-connected layers. For image classification we trained the network that consists of the following layers:

- Flatten layer—to resize the images
- Dense (Fully-connected) layer with 512 neurons with ReLU activation function
- Dense layer with 10 neurons and Softmax activation function to classify 10 classes

The network that was used for the Reuters dataset (text classification) consists of the following layers:

- Two Dense layers with 64 neurons each, with ReLU activation function
- One Dense layer with 46 neurons and Softmax activation function for 46 classes

A regular deep learning methodology consists of following steps: (1) data preprocessing, (2) DNN architecture implementation and configuration, (3) learning and fitting the model to the data. The last stage is mainly based on the cost function, as it’s behaviour depends on a set of training parameters (such as number of epochs, learning rate or batch size). In our experiment to enable the comparison of optimization algorithms the data sets including training and testing parts as well as labels remain fixed. Only training parameters are the subject of optimisation.

**Figure 3:**
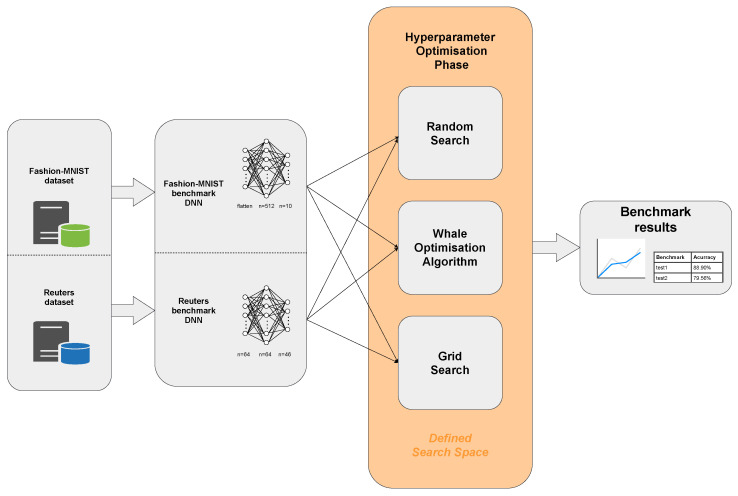
Benchmark diagram for deep neural network hyperparameter optimisation.

Our search space is a *n*-dimensional grid, where *n* corresponds to the number of parameters. For each task, we decide which parameters are important and belong to the search space constrains. For the first few experiments, we chose pairs of parameters and optimised them as a 2-dimensional search space. Then, we adjusted the algorithm to work in a 3-dimensional space and optimised a set of three parameters (due to the fact that optimization problems in the DNN field rarely remain in 2d search space). As suggested in [8] the algorithm can theoretically be further extended to *n*-dimensions. To achieve this, we have refactored solution vectors as well as whole mathematical model (see Section 2.1 to operate in 3-dimensional space. Our 3D-WOA algorithm remained similar to the original open source one provided by the author (see [23]) and extended to utilize 3D vectors. Finally, runtime and benchmark functions APIs have been adjusted to work with 3-dimensional space. The methodology of the conducted WOA-based deep neural network hyperparameter optimisation research has been presented in Figure 3.

### 2.3. WOA Implementation for Deep Learning Approach

Whale Optimisation Algorithm is available as open source on author’s website [23]. A set of parameters has been enabled and can be adjusted to a specific problem. The most important include cost function, constrains, number of generations, number of solutions per generation, and parameters *a* and *b* (see Section 2.1.2).

Firstly, in order to optimize the cost function, a separate class with optimization function has been defined. It takes training parameters as arguments and runs fitting operation with those parameters. To evaluate the models’ performance and compare WOA with other popular algorithms, we have applied k-fold cross-validation procedure with manually adjusted parameter k equal to 3. We decided to base our optimisation only on cross-validation accuracy metrics which is returned from the function and serves as a cost function value.

Parameters, that have to be carefully adjusted when using Whale Optimisation Algorithm are those responsible for the search space and constrains. The problem (and at the same time limitation) of the algorithm we had to cope with was the fact that WOA works only in continuous space whereas parameters of the neural network (e.g., epoch or batch size) have discrete values. Therefore we had to adapt the WOA to solve discrete optimisation problem. To achieve it, discretization has been performed inside the main body of the algorithm class. All parameters have been rounded to the nearest integer at the stage of solution vector generation—new solutions generated by the WOA are immediately converted to the nearest integer and returned to the main body of the algorithm already as discrete values. Moreover, in some cases lookup table encoding has been used—e.g., for coding optimiser selection (specific integer number corresponds to a specific optimizer). What is more, by applying appropriate constraints to the solutions, we move in a search space applicable for a given problem. Exact search spaces have been described in Section 3.

Finally, the number of generations and solutions per generation has been set. The first parameter describes how many iterations the program should run and the second specifies the number of agents. Both parameters have great impact on the obtained results: the higher they are the more swarm-based the algorithm becomes. However, more agents also increases computational complexity. Each new generation or agent requires a deep neural network to be trained from scratch. To compare the computation time we assumed, that the number of generations multiplied by the number of solutions per generation, should be similar to the number of iterations in non-heuristic algorithms. Parameters *a* and *b* have been set to their default values.

### 2.4. Improving the Computational Speed

The Whale Optimisation Algorithm was primarily designed for optimising mathematical functions, which are not computationally demanding. Therefore the algorithms implementation often calculates the cost function value in a specific search space, for example to compare current solution with the best one, or rank solutions achieved by each agent in a generation. In our case, as the training phase is long and complex it was necessary to adapt functions to our needs.

In order to reduce the computational operations we have implemented a list for storing the calculated values. During operation the algorithm checks whether the value in a specific point of search space has already been memorized. This method vastly shortens the time and reduces the random effects of neural network training.

## 3. Experiments

We tested our solution on widely-used multi-class classification problems including MNIST, Fashion MNIST and Reuters datasets. As originally the algorithm was available only for two dimensions, we created following parameter sets: (1) number of epochs and batch size and (2) number of epochs and optimizer. The range of parameters was as follows:

- Batch Size in range 10–500 in continuous search and [8, 16, 32, 64, 128, 256, 512] in a discrete one,
- Epochs in range 5–50 in continous search and [5, 10, 15, 20, 25, 30, 35, 40, 45, 50] in a discrete one,
- Optimzer selected from SGD, RMSProp, Adam, Adamax, Adagrad, Adadelta.

**Figure 4:**
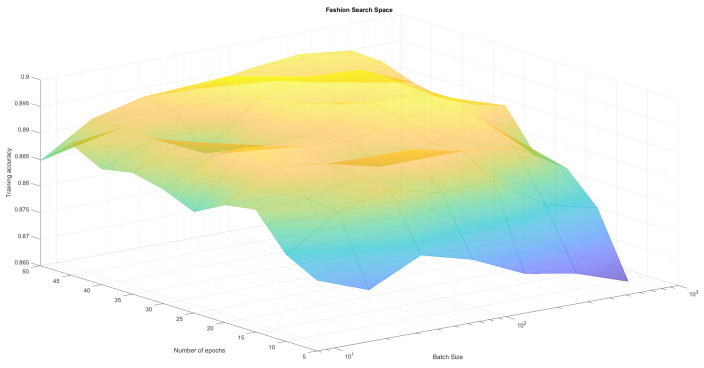
Epoch and batch search space for Fashion-MNIST classification. We cannot observe one clear maximum, but there are areas that should be avoided.

**Figure 5:**
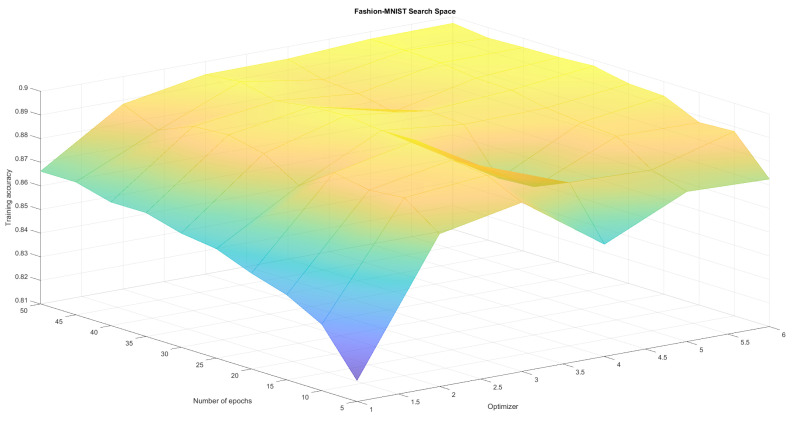
Epoch and optimizer search space for Fashion-MNIST classification. We cannot observe one clear maximum, but for certain optimizers the performance drops.

**Figure 6:**
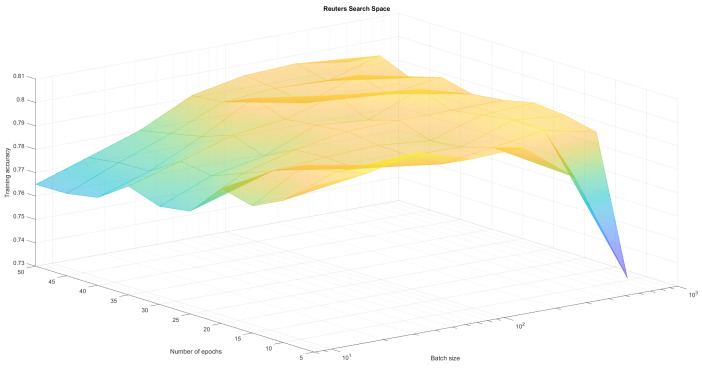
Epoch and batch search space for Reuters classification. We cannot observe one clear maximum, but there are areas that should be avoided.

**Figure 7:**
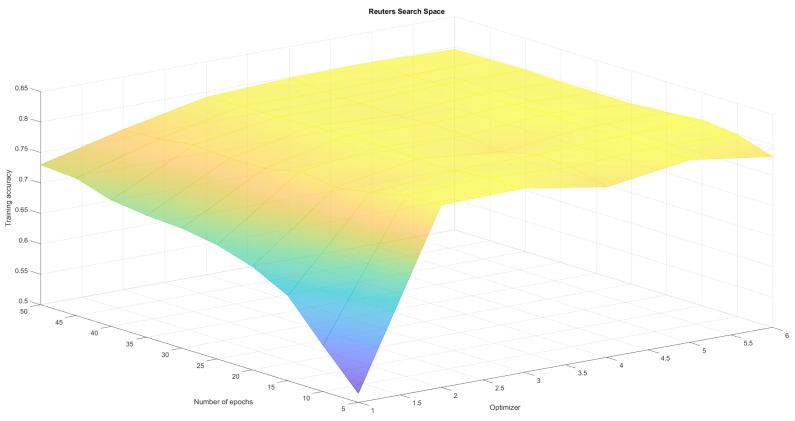
Epoch and optimizer search space for Reuters classification. We cannot observe one clear maximum, but for certain optimizers the performance drops.

The neural network regardless of the training parameters achieved comparable results for the MNIST dataset which is considered to be one of the simplest. This confirmed the correct operation and implementation of the algorithm but it was therefore hard to observe differences in results. As a result we created four search spaces in which we conducted optimization experiments:

- Fashion-MNIST classification with variable number of epochs and batches (set Adam optimizer)—see Table 1, Figure 4
- Fashion-MNIST classification with variable number of epochs and optimizers (set batch 128)—see Table 2, Figure 5,
- Reuters classification with variable number of epochs and batches (set Adam optimizer)—see Table 3, Figure 6,
- Reuters classification with variable number of epochs and optimizers (set batch 128)—see Table 4, Figure 7.

To create a reference point for our research, we run those four optimisation experiments (two pairs of parameters for two different problems) using classical methods Grid Search and Random Search algorithm. The first one does an exhaustive search over a fixed parameter grid. It allowed us to find local maxima, as well as observe full solution space and visualize it in Figure 4, Figure 5, Figure 6 and Figure 7. Random Search method, which selects *n* random sets of parameters from established range was used for comparison. It can achieve at least average solutions with no meta-heuristic. We run RandomSearchCV with 20 random solutions, to compare it’s computation time with WOA, which was run for 4 generations with 5 solutions each. Reference results have been presented in Table 1, Table 2, Table 3 and Table 4.

Secondly, we have tested our 3D-WOA solution on the same multi-class classification problems, including Fashion MNIST and Reuters datasets. Now, with 3-dimensional search space available, we conducted two experiments:

- Fashion-MNIST classification with variable number of epochs, batches and optimizers—see Table 5
- Reuters classification with variable number of epochs, batches and optimizers—see Table 6

Finally, we have added additional parameter *total time* describing the computation time for each experiment using the computational resources given in Section 3.1. Total time has been computed using the most popular for this purpose *time* Python module. It is based on a way that Unix systems calculate so called *epoch time* (number of seconds that have elapsed since 1 January 1970). This timestamp was saved with the launch and the termination of each algorithm, and then to calculate the total time, the difference between these values has been calculated. The accuracy of the time measured this way is on the order of one second but because of legibility and the order of magnitude of measured values, we decided to round them to one minute. What is worth mentioning, the Whale Optimisation Algorithm often achieves similar or better accuracy results than the Grid Search or Random Search with shorter execution times, which can be seen especially in Table 3 and Table 4.

As can be observed in Figure 4, Figure 5, Figure 6 and Figure 7 the search space has local minima as well as saddle points which makes it hard to spot the global maximum. We also have to keep in mind, that training every neural network is susceptible to a certain level of randomness, due to factors such as initial weights, batch distribution, train-test split or vanishing gradient. Because of that, we avoid choosing one global maximum, which may vary even when running a full Grid Search several times. Therefore, we assumed that the algorithm works correctly if it finds one or more local maximum, that is in top-4 best solutions. Our method returns those top-4 sets of parameters to allow the user to choose the best one for them (for example batch according to available memory).

Table 1, Table 2, Table 3, Table 4, Table 5 and Table 6 prove that neural network training is susceptible to a certain level of randomness. There are even examples of same sets of parameters with slightly different accuracy. However, the fact that similar results appear in top places indicates the existence some form of maximum. From tables we can also read, that each method behaves correctly—GridSearch top4 results are quite stable and high. Random Search top4 accuracy are often more diverse, ranging from much worse than grid search to sometimes even better due to training randomness. This method is much less predictable, but much faster. WOA, on the other hand continuously improves in each generation, offering an equally good and stable alternative to non-heuristic methods. Finally, in search space plots there are visible pitfalls—parameter sets, that result in drastic accuracy drop. All three methods managed to at least avoid those areas completely. By analysing full results, generated by GridSearch, we can observe that about half of the parameter sets produces significantly worse results (with a performance drop of more than 10%) compared to Top4. Furthermore, some optimizers (like Adadelta or Adagrad), failed to converge at all for some sets of batches and epochs. Although the fastest algorithm is the random search, we can only guarantee that it won’t randomly generate those pitfalls by increasing the number of random sets. Furthermore, random search does not really give an explanation about why this parameter set is optimal. In the applications where the explainability of each stage of a method is expected WOA (or other bio-inspired algorithm) might be a good replacement.

In Table 5 and Table 6 we present results from 3D-WOA algorithm. We can conclude that the method behaves correctly and Top4 results are comparable to those achieved by running two separable experiments (see Table 1, Table 2, Table 3 and Table 4). One that stands out in this experiment is its total time which is fairly long.

### 3.1. Software and Hardware

All experiments were executed using Python 3.7 programming language with Keras 2.3 library and trained on a NVIDIA RTX 2070 Super GPU (8 GB) with 48 GB RAM and Intel i7 Processor. The code of WOA was adapted based on the implementation provided by the author of the original paper. Code of our work is available publicly on GitLab under the following link: https://gitlab.com/piekarski.michal/whaleoptimisationalgorithm, accessed on 16 November 2021.

## 4. Discussion

Analysis of the obtained results and search spaces clearly show that Whale Optimisation Algorithm has some potential in hyperparameters tuning. The solutions generated by WOA seem to be promising as they achieve better results mostly in shorter time than the simpler methods. Future research will focus on testing this solution on more complex deep learning problems, with more varied local extrema in search space.

In our opinion one of the main problems encountered during this study was establishing solution space for WOA. All examples implemented by Mirjalili in the algorithm’s original paper were mathematical functions. While sometimes very complex, they were all calculable in continuous space within a short time and the algorithm’s steps were designed for that. In our task, we often have to choose between non-numerical parameters, such as optimizers. In that case we had to implement dictionaries coding words as numbers. In other (like epochs) we had to round real numbers to integers. The optimization algorithm, designed to work in continuous space, sometimes struggle to find an optimum because of this approximations. We strongly suggest, that any researchers wishing to utilise Whale Optimisation Algorithm for hyperparameters tuning, should take this facts into consideration and carefully think about transfer function they use for discretisation.

Other bio-inspired methods, such as genetic algorithm, with non-continuous operations like mutations, should be also very powerful in this field of hyperparameters optimization [24]. We see a huge potential in swarm-based methods as well and plan to work on improving our method in future. Recently published papers, that also propose a discrete versions of Whale Optimisation Algorithm (DWOA) are promising [25,26] and supports this direction. Unfortunately, they were not available as an open source, so we could not compare transfer functions used in those papers with our own discrete implementation.

## 5. Conclusions

In this research we managed to adapt Whale Optimisation Algorithm to Deep Learning purpose. We proved that training a deep neural network can be treated in the same way as any other cost function and optimised using swarm-based method. We implemented a transfer function that maps a discrete set of parameters into a search space suitable for WOA. We also managed to extend the basic 2D version of the algorithm to work in a 3D search space. Our solution was able to correctly find the good set of training hyperparameters in shorter time than Grid Search method, but comparable to much simpler Random Search. In the future we will work on better discretisation and testing on datasets with more varied local extrema in search spaces.

To the best of our knowledge this is the first solution that deals with the problem of neural network training hyperparameters tuning, using metaheuristic swarm-based Whale Optimisation Algorithm. While the method is not perfect, we believe that it will become a basis for future researchers wanting to utilise WOA, as we expect it to be easily applied to other tasks. We hope, that this paper might give them some useful suggestions about the limitations they need to consider and the possible changes that have to be implemented to adapt WOA for deep learning (for example a proper transfer function for discretisation).

## Figures and Tables

**Table 1:** Fashion MNIST, epoch (0–50), discrete batch, Adam optimiser.

Batch	Epoch	Accuracy [%]	Total Time
WOA Best Each Generation			
64	23	89.11	
64	23	89.31	119 min
64	27	89.34	
**64**	**27**	**89.49**	
**Random Search**			
64	45	89.49	
128	25	89.40	63 min
512	45	89.23	
16	30	89.21	
**Grid Search**			
128	35	89.68	
64	45	89.57	220 min
64	20	89.45	
256	45	89.44	

**Table 2:** Fashion MNIST, epoch (0–50), discrete optimiser, batch 128.

Optimiser	Epoch	Accuracy [%]	Total Time
WOA Best Each Generation			
adam	43	89.19	
adamax	50	89.72	33 min
adamax	50	89.79	
**adamax**	**50**	**89.84**	
**Random Search**			
adagrad	50	89.83	
adagrad	45	89.74	21 min
adamax	20	89.53	
adagrad	40	89.52	
**Grid Search**			
adamax	45	89.88	
adam	40	89.49	62 min
adamax	30	89.35	
adam	30	89.06	

**Table 3:** Reuters, epoch (0–50), discrete batch, Adam optimiser.

Batch	Epoch	Accuracy [%]	Total Time
WOA Best Each Generation			
128	12	80.00	
128	12	80.10	8 min
256	13	80.12	
**512**	**12**	**80.33**	
**Random Search**			
512	15	80.07	
16	5	80.04	23 min
32	10	80.03	
32	20	79.79	
**Grid Search**			
64	15	80.17	
256	15	79.62	80 min
256	50	79.56	
16	15	79.01	

**Table 4:** Reuters, epoch (0–50), discrete optimiser, batch 128.

Optimiser	Epoch	Accuracy [%]	Total Time
WOA Best Each Generation			
adamax	20	79.89	
adamax	31	80.54	7 min
adamax	20	80.55	
**adamax**	**31**	**80.60**	
**Random Search**			
adagrad	25	80.13	
adadelta	25	80.09	12 min
adagrad	20	80.02	
adagrad	40	79.93	
**Grid Search**			
adam	5	80.29	
adamax	40	79.70	36 min
adagrad	25	79.53	
adamax	45	79.33	

**Table 5:** Fashion MNIST 3D search space, epoch (0–50), discrete optimiser, discrete batch.

Optimiser	Epoch	Batch	Accuracy [%]	Total Time
WOA Best Each Generation				
**adamax**	**30**	**512**	**89.75**	
SGD	44	8	88.65	
SGD	50	512	89.30	1149 min
adamax	30	512	89.50	
**Random Search**				
adamax	40	128	89.41	
adam	40	32	89.19	38 min
adam	50	32	89.18	
rmsprop	50	128	88.90	
**Grid Search**				
adamax	45	16	89.88	
adamax	50	32	89.85	620 min
adamax	35	32	89.83	
adamax	45	64	89.75	

**Table 6:** Reuters 3D search space, epoch (0–50), discrete optimiser, discrete batch.

Optimiser	Epoch	Batch	Accuracy [%]	Total Time
WOA Best Each Generation				
adam	20	32	76.59	
adamax	23	8	78.65	160 min
adamax	29	512	79.30	
**adamax**	**29**	**512**	**79.46**	
**Random Search**				
adamax	45	256	80.36	
adam	10	256	80.24	10 min
adamax	10	8	80.23	
adam	20	512	80.01	
**Grid Search**				
rmsprop	5	16	80.50	
adamax	20	32	80.49	200 min
adamax	15	64	80.48	
adamax	25	128	80.41	

## Data Availability

Datasets used in this research were downloaded from tf.keras.datasets module, as explained on website: https://keras.io/api/datasets/.
